# Case report: Cutaneous pseudolymphoma caused by a *Leishmania infantum* infection in a patient treated with anti-TNF antibody for plaque psoriasis

**DOI:** 10.3389/fmed.2022.1055703

**Published:** 2022-12-02

**Authors:** Sarah Scholl, Daniel Schuster, Kristin Technau-Hafsi, Katarina Stete, Siegbert Rieg, Annette M. May, Christian Bogdan, Franziska Schauer

**Affiliations:** ^1^Department of Dermatology, Medical Center – University of Freiburg, Faculty of Medicine, University of Freiburg, Freiburg, Germany; ^2^Division of Infectious Diseases, Department of Medicine II, University Hospital Freiburg, Freiburg, Germany; ^3^Institute for Clinical Pathology, Medical Center – University of Freiburg, Faculty of Medicine, University of Freiburg, Freiburg, Germany; ^4^Institute for Dermatohistology, Pathology, Molecular Pathology Prof. Dr. Helmut Laaff, Freiburg, Germany; ^5^Mikrobiologisches Institut–Klinische Mikrobiologie, Immunologie und Hygiene, Universitätsklinikum Erlangen, Friedrich-Alexander-Universität (FAU) Erlangen-Nürnberg, Erlangen, Germany; ^6^Medical Immunology Campus Erlangen, Friedrich-Alexander-Universität (FAU) Erlangen-Nürnberg, Erlangen, Germany

**Keywords:** skin pseudolymphoma, cutaneous leishmaniasis, anti-TNF therapy, adalimumab, skin ulceration

## Abstract

For psoriasis, which affects up to 2% of the population and adalimumab is approved from the age of 4 years. Here, we present a middle-aged Italian man with long-term history of plaque psoriasis and psoriasis arthropathica and adalimumab therapy. He developed ulcers or nodules within the psoriatic plaques, resembling cutaneous infection with *Leishmania infantum*. TNF and other cytokines such as IL-12 and IFN-γ are central in the early control of the infection. Discontinuation of the anti-TNF-treatment resolved the infection without specific therapy.

## Introduction

Tumor necrosis factor (TNF) inhibitors have been in use for a variety of diseases since more than 20 years. Adalimumab is a human recombinant monoclonal immunoglobulin G1 antibody, which binds to both soluble and tissue-bound TNF, followed by its inactivation and degradation (1, 2). As TNF not only acts as a proinflammatory cytokine during chronic inflammatory diseases, but also is critical for the control of various intracellular pathogens, TNF antagonists are capable of causing reactivation of certain viral, bacterial, protozoan, or fungal infections (3, 4). For psoriasis, which affects up to 2% of the population, adalimumab is approved from the age of 4 years under certain conditions (5). Here, we present a middle-aged Italian man with long-term history of plaque psoriasis and psoriasis arthropathica and adalimumab therapy. Following stays in his home country, he developed ulcers or nodules within the psoriatic plaques, resembling cutaneous pseudolymphoma in histopathology. Parasitological diagnostics revealed a cutaneous infection with *Leishmania infantum*, which clinically resolved without specific therapy following discontinuation of the anti-TNF-treatment.

## Case description

A 42-year-old Italian patient with a 20-year history of plaque psoriasis and psoriasis arthropathica presented to our hospital with numerous ulcerations or nodules within his psoriatic lesions that had appeared 6 months earlier. He had been treated with subcutaneous methotrexate (25 mg per week) for the past 9 years and adalimumab (40 mg every other week) for the past 7 years. Topical treatment included halometasone 0.05% plus triclosan 1% cream and calcipotriol plus betamethasone dipropionate foam in the weeks before hospitalization.

**FIGURE 1:**
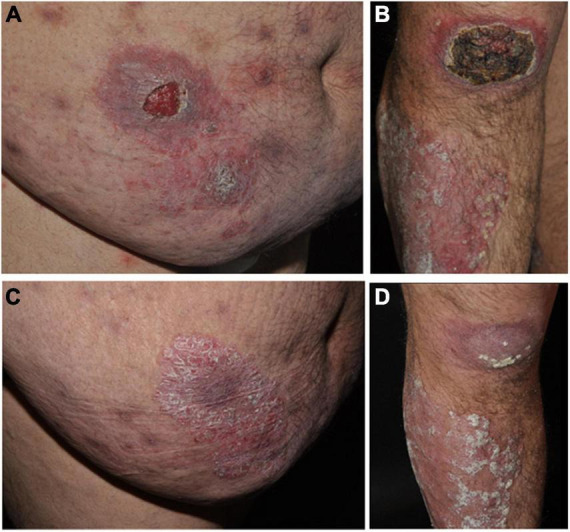
Panel **(A)** Ulceration (4 cm in diameter) within a psoriatic plaque on the right abdomen of the obese patient receiving anti-TNF therapy (adalimumab). Panel **(B)** Up to 10 cm pseudolymphomatous tumor surrounded by a psoriatic plaque on the right knee of the patient caused by cutaneous *Leishmania infantum* infection under anti-TNF therapy and erythematosquamous plaque on the right tibia. Panel **(C)** Erythematosquamous plaque on the right abdomen of the patient after cessation of adalimumab therapy. Panel **(D)** Erythematosquamous plaque on the right knee and shin of patients after cessation of adalimumab therapy.

The obese patient (BMI 39 kg/m^2^) showed multiple psoriatic lesions characterized by erythemato-squamous plaques disseminated over the integument (PASI 16.6). Moreover, the patient showed erosive and partly ulcerating plaques and nodules, some of them covered by distinct brown crusts, at the right forearm, right knee, left flank and the abdomen (Figures 1A,B). Physical examination showed no pathological findings. There was no lymphadenopathy.

**FIGURE 2:**
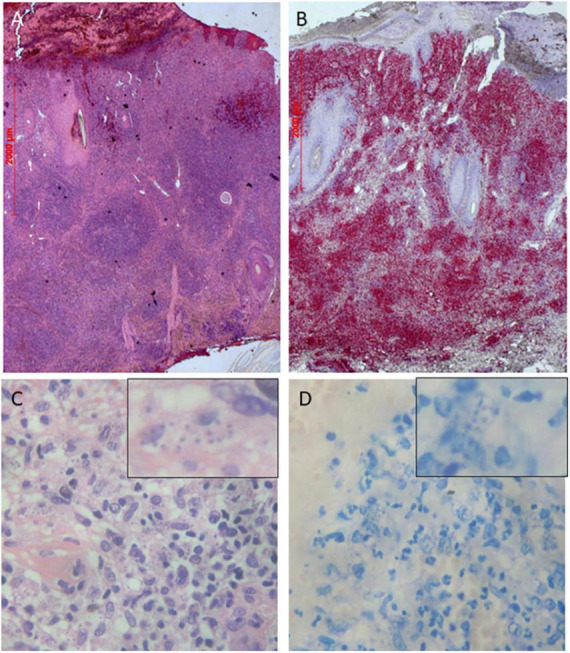
Panel **(A)** Histopathology shows a dense nodular, perivascular, periadnexal and interstitial infiltration of mainly T-cells and lymphocytes (Hematoxylin-eosin stain, ×25) Panel **(B)** Immunohistochemistry with CD3 antibodies (×25). Histopathology shows multiple intracellular amastigotes with peripheral nuclei and kinetoplasts in Hematoxylin-eosin stain Panel **(C)** and in Giemsa stain Panel **(D)** (×400 magnification each).

The histopathology of an externally performed lesion biopsy reported dermal T-cell infiltrates without evidence repeated of malignancy. Two repeat biopsies and histologies from morphologically different lesions on the right forearm and right flank revealed an interface dermatitis with pseudolymphomatous infiltrates, thickening of the epidermis, follicular hyperparakeratosis, and perivascular, periadnexal, superficially accentuated lymphocytic infiltrates with a preponderance of T cells (Figures 2A,B). Tissue clonality analyses demonstrated polyclonal IgH and TCR gamma chain gene expression, thus excluding lymphoma. Direct immunofluorescence did not detect any IgG, IgM, IgA, or C3 depositions. Syphilis serology, carried out due to numerous plasma cells in the tissue, yielded negative results. Moreover, an HIV infection, borreliosis and tuberculosis were ruled out by negative antibody or PCR tests. A serum sample was negative for antibodies against (extractable) nuclear antigens. Complement factors were within normal ranges. In hematoxylin-eosin (Figure 1C), Giemsa (Figure 1D) and Feulgen stains, intracellular *Leishmania* amastigotes with kinetoplasts were seen, which were identified by culture, miniexon PCR and restriction fragment length polymorphism-analysis as *L. infantum*. The patient also had a positive anti-Leishmania serology (maximum titer detected by indirect immunofluorescence using viable *L. major* promastigotes was 1:1,600). Since the patient originated from and repeatedly visited southern Italy, it is likely that he acquired the infection during his temporary stays.

**FIGURE 3:**
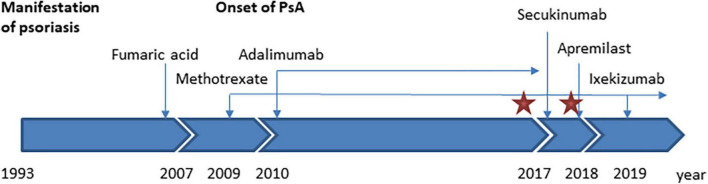
Time course of the patient’s medical history, treatment, and *Leishmania infantum* infection. The red star marks the time of detection of *L. infantum* in skin biopsies. PsA, psoriasis arthropathica.

The therapy with adalimumab was stopped, while treatment with methotrexate was continued (Figure 2). Interestingly, the ulcerations already started to heal under intensive topical treatment with salicylic acid, dithranol, and clobetasol propionate 0.05% ointment. Visceral involvement was excluded based on clinical parameters and sonography of liver and spleen. Therefore, we pursued a watch-and-wait strategy without specific therapy for leishmaniasis. The ulcerative skin lesions, which were clinically compatible with cutaneous leishmaniasis, completely regressed. After switching the systemic treatment to secukinumab (anti-IL-17A) or apremilast, the psoriatic lesions remained unaltered, but finally improved with ixekizumab (anti-IL-17A). During regular medical follow-up for almost 5 years the clinical condition of the patient remained stable (Figure 3).

## Discussion

Cutaneous pseudolymphoma (CPSL) represents a heterogeneous group of benign lymphoproliferative reactions clinically and histologically mimicking lymphomas. Depending on the etiology, it can be subdivided into CPSL caused by infections (e.g., bacteria, viruses, parasites), drugs (e.g., anticonvulsants), foreign substances (e.g., vaccinations, tattoos), or other reasons (6). CPSL triggered by drugs (29%) and tattoos (26%) are the most frequent causes (6).

In our case, the patient’s pseudolymphomas and ulcerations were due to an infection with *L. infantum* during immunosuppressive therapy for the treatment of psoriasis with adalimumab and methotrexate. *Leishmania* spp. are flagellated protozoan parasites that are transmitted by bites of sand fly vectors. Infections are endemic in almost 90 countries, including southern Europe and the entire Mediterranean coast (7, 8). Given the high number of positive leishmanin skin tests in Alicante, Spain (50% of adults, 10% of children), individuals may also be asymptomatic carriers of the parasite associated with transient or intermittent parasitemia (9, 10). There are several case reports describing pseudolymphoma and skin ulcerations as the cutaneous manifestation of leishmaniasis (11, 12) and infections are sometimes associated with anti-TNF therapy (3, 4). Cutaneous pseudolymphoma reactions can also be triggered by anti-TNF treatment in the absence of specific infections (8, 13–15). *Leishmania* spp. primarily infect and replicate within mononuclear phagocytes. Both the innate and adapted immune system are required for control. TNF and other cytokines such as IL-12 and IFN-γ are central in the early control of the infection. TNF and IFN-γ are responsible for the induction of leishmanicidal activity of macrophages, which is characterized by the production of reactive oxygen species, nitric oxide and metabolic changes of infected cells (16). The risk of *Leishmania* infections is therefore increased for individuals from endemic areas, who are treated with one or more immunosuppressive agents (e.g., glucocorticosteroids, methotrexate, azathioprine, or TNF inhibitors) (8, 17). *L. infantum* can cause cutaneous or visceral leishmaniasis, the latter being more frequent during childhood or in patients with poor immune status (e.g., HIV infection).

The anti-leishmanial treatment depends on the causative species, the clinical manifestation (cutaneous, mucocutaneous, or visceral manifestation) and the presence of underlying medical conditions (18). Bosch-Nicolau et al. (17) reported on 59 infected patients from the Mediterranean basin and found a more benign natural course in cutaneous leishmaniasis caused by *L. infantum* with spontaneous healing and local wound care after discontinuation of TNF inhibitors, especially if lesions were below 5 cm in diameter and in localized, non-disabling areas. On the other hand 14% of their treated cases relapsed despite etiological therapy. Although our patient showed complex features of cutaneous infection without mucosal or visceral involvement, we only omitted TNF inhibitor therapy and implemented psoriasis specific topical treatment. The role of dithranol and its anti-proliferative and anti-inflammatory effects in cutaneous leishmaniasis remains unclear. The cytokines IL-17, IL-22, and IL-23 play an important role in the development of psoriasis and anti-IL-17A therapy significantly improved our patient’s skin outcome (19). To date, no cases of leishmanial infection have been published in patients treated with anti-IL-17A antibodies (secukinumab, ixekizumab) or phosphodiesterase 4-inhibitors (apremilast).

Data even suggest for a protective effect of TH17 cells in controlling tissue parasitism (20).

Our case highlights the need for increased awareness for leishmaniasis in patients from endemic areas, who are treated with immunosuppressants such as TNF inhibitors. Serological screening can be considered to detect latent *Leishmania* infections, although it is of limited value in localized (muco-) cutaneous infections. Repeated skin biopsies should be performed if new atypical skin lesions arise. Histologically diagnosed pseudolymphomatoid reactions require further diagnostic tests to rule out causes such as leishmaniasis and other infections.

## Data availability statement

The raw data supporting the conclusions of this article will be made available by the authors, without undue reservation.

## Ethics statement

Ethical review and approval was not required for the study on human participants in accordance with the local legislation and institutional requirements. The participants provided their written informed consent to participate in this study.

## Author contributions

FS, DS, KS, and SR took care for the patient over the years. KT-H, AMM, and CB performed the diagnostics and were involved in making the diagnosis. SS drafted the manuscript. FS and CB edited the manuscript. All authors read and agreed to the published version of the manuscript.

## References

[1] Gordon KB Langley RG Leonardi C Toth D Menter MA Kang S Clinical response to adalimumab treatment in patients with moderate to severe psoriasis: double-blind, randomized controlled trial and open-label extension study. *J Am Acad Dermatol.* (2006) 55:598–606. 10.1016/j.jaad.2006.05.027 17010738

[2] Kaymakcalan Z Sakorafas P Bose S Scesney S Xiong L Hanzatian DK Comparisons of affinities, avidities, and complement activation of adalimumab, infliximab, and etanercept in binding to soluble and membrane tumor necrosis factor. *Clin Immunol.* (2009) 131:308–16. 10.1016/j.clim.2009.01.002 19188093

[3] Bogdan C. Leishmaniasis in rheumatology, haematology and oncology: epidemiological, immunological and clinical aspects and caveats. *Ann Rheum Dis.* (2012) 71 (Suppl. 2):i60–6. 10.1136/annrheumdis-2011-200596 22460140

[4] Baddley JW Cantini F Goletti D Gómez-Reino JJ Mylonakis E San-Juan R Study group for infections in compromised hosts (ESGICH) consensus document on the safety of targeted and biological therapies: an infectious diseases perspective (Soluble immune effector molecules [I]: anti-tumor necrosis factor-α agents). *Clin Microbiol Infect.* (2018) 24 (Suppl. 2):S10–20. 10.1016/j.cmi.2017.12.025 29459143

[5] Papp K Thaçi D Marcoux D Weibel L Philipp S Ghislain P-D Efficacy and safety of adalimumab every other week versus methotrexate once weekly in children and adolescents with severe chronic plaque psoriasis: a randomised, double-blind, phase 3 trial. *Lancet.* (2017) 390:40–9. 10.1016/S0140-6736(17)31189-3 28478975

[6] Mitteldorf C Kempf W. Cutaneous pseudolymphoma-A review on the spectrum and a proposal for a new classification. *J Cutan Pathol.* (2020) 47:76–97. 10.1111/cup.13532 31237707

[7] World Health Organization. *Leishmaniasis.* (2022). Available onlinbe at: https://www.who.int/health-topics/leishmaniasis#tab=tab_1 (accessed September 18, 2022)

[8] Català A Roé E Dalmau J Pomar V Muñoz C Yelamos O Anti-tumour necrosis factor-induced visceral and cutaneous leishmaniasis: case report and review of the literature. *Dermatology.* (2015) 230:204–7. 10.1159/000370238 25633623

[9] Moral L Rubio EM Moya M. A leishmanin skin test survey in the human population of l’Alacantí region (Spain): implications for the epidemiology of *Leishmania infantum* infection in southern Europe. *Trans R Soc Trop Med Hyg.* (2002) 96:129–32. 10.1016/s0035-9203(02)90278-6 12055798

[10] Michel G Pomares C Ferrua B Marty P. Importance of worldwide asymptomatic carriers of *Leishmania infantum* (L. *chagasi*) in human. *Acta Trop.* (2011) 119:69–75. 10.1016/j.actatropica.2011.05.012 21679680

[11] Recalcati S Vezzoli P Girgenti V Venegoni L Veraldi S Berti E. Cutaneous lymphoid hyperplasia associated with *Leishmania panamensis* infection. *Acta Derm Venereol.* (2010) 90:418–9. 10.2340/00015555-0893 20574614

[12] Flaig MJ Rupec RA. Cutaneous pseudolymphoma in association with Leishmania donovani. *Br J Dermatol.* (2007) 157:1042–3. 10.1111/j.1365-2133.2007.08129.x 17714564

[13] Imafuku S Ito K Nakayama J. Cutaneous pseudolymphoma induced by adalimumab and reproduced by infliximab in a patient with arthropathic psoriasis. *Br J Dermatol.* (2012) 166:675–8. 10.1111/j.1365-2133.2011.10607.x 21910704

[14] Safa G Luce K Darrieux L Tisseau L Ortonne N. Erythrodermic CD8+ pseudolymphoma during infliximab treatment in a patient with psoriasis: use of cyclosporine as a rescue therapy. *J Am Acad Dermatol.* (2014) 71:e149–50. 10.1016/j.jaad.2014.05.042 25219741

[15] Carvalhana S Gonçalves A Velosa J. Cutaneous pseudolymphoma in a patient with Crohn’s disease under infliximab: first report. *J Clin Gastroenterol.* (2016) 50:436–7. 10.1097/MCG.0000000000000513 26974759

[16] Bogdan C. Macrophages as host, effector and immunoregulatory cells in leishmaniasis: impact of tissue micro-environment and metabolism. *Cytokine X.* (2020) 2:100041. 10.1016/j.cytox.2020.100041 33604563 PMC7885870

[17] Bosch-Nicolau P Ubals M Salvador F Sánchez-Montalvá A Aparicio G Erra A Leishmaniasis and tumor necrosis factor alpha antagonists in the Mediterranean basin. A switch in clinical expression. *PLoS Negl Trop Dis.* (2019) 13:e0007708. 10.1371/journal.pntd.0007708 31469834 PMC6742442

[18] Boecken G Sunderkötter C Bogdan C Weitzel T Fischer M Müller A [Diagnosis and therapy of cutaneous and mucocutaneous Leishmaniasis in Germany]. *J Dtsch Dermatol Ges.* (2011) 8:1–51. 10.1111/j.1610-0379.2011.07820.x 22050890

[19] Murdaca G Colombo BM Puppo F. The role of Th17 lymphocytes in the autoimmune and chronic inflammatory diseases. *Intern Emerg Med.* (2011) 6:487–95. 10.1007/s11739-011-0517-7 21258875

[20] Murdaca G Gerosa A Paladin F Petrocchi L Banchero S Gangemi S. Vitamin D and microbiota: is there a link with allergies? *Int J Mol Sci.* (2021) 22:4288. 10.3390/ijms22084288 33924232 PMC8074777

